# Coronary CT angiography-based estimation of myocardial perfusion territories for coronary artery FFR and wall shear stress simulation

**DOI:** 10.1038/s41598-021-93237-1

**Published:** 2021-07-05

**Authors:** Yu-Fang Hsieh, Chih-Kuo Lee, Weichung Wang, Yu-Cheng Huang, Wen-Jeng Lee, Tzung-Dau Wang, Cheng-Ying Chou

**Affiliations:** 1 Department of Biomechatronics Engineering, National Taiwan University, Taipei, 106 Taiwan; 2 Department of Internal Medicine, National Taiwan University Hospital Hsin-Chu Branch, Hsinchu, 300 Taiwan; 3 Institute of Applied Mathematical Sciences, National Taiwan University, Taipei, 106 Taiwan; 4 Department of Medical Imaging, National Taiwan University Hospital, Taipei, 100 Taiwan; 5 Cardiovascular Center and Divisions of Cardiology and Hospital Medicine, Department of Internal Medicine, National Taiwan University Hospital, Taipei, 100 Taiwan

**Keywords:** Biophysics, Cardiology

## Abstract

This study aims to apply a CCTA-derived territory-based patient-specific estimation of boundary conditions for coronary artery fractional flow reserve (FFR) and wall shear stress (WSS) simulation. The non-invasive simulation can help diagnose the significance of coronary stenosis and the likelihood of myocardial ischemia. FFR is often regarded as the gold standard to evaluate the functional significance of stenosis in coronary arteries. In another aspect, proximal wall shear stress ($\mathrm{{WSS}_{prox}}$) can also be an indicator of plaque vulnerability. During the simulation process, the mass flow rate of the blood in coronary arteries is one of the most important boundary conditions. This study utilized the myocardium territory to estimate and allocate the mass flow rate. 20 patients are included in this study. From the knowledge of anatomical information of coronary arteries and the myocardium, the territory-based FFR and the $\mathrm{{WSS}_{prox}}$ can both be derived from fluid dynamics simulations. Applying the threshold of distinguishing between significant and non-significant stenosis, the territory-based method can reach the accuracy, sensitivity, and specificity of 0.88, 0.90, and 0.80, respectively. For significantly stenotic cases ($\mathrm{FFR}_{m}$ $\le$ 0.80), the vessels usually have higher wall shear stress in the proximal region of the lesion.

## Introduction

Cardiovascular disease is the top leading cause of death worldwide. Total deaths associated with cardiovascular diseases had increased by 21.1% from 2007 to 2017 and claimed 17.8 million deaths in 2017^1^. The most common type of cardiovascular disease is atherosclerotic coronary artery disease (CAD), which might lead to narrowing or obstruction of the coronary artery and subsequent myocardial ischemia. Aside from causing lumen stenosis, the lipid deposits into the arterial wall and forms atherosclerotic plaques which could cause catastrophic and emergent cardiovascular events. An increase in blood pressure or mechanical stress can cause a vulnerable plaque rupture and subsequent coronary occlusion, resulting in myocardial necrosis. This is the so-called heart attack or myocardial infarction.

Fractional flow reserve (FFR) expresses the maximal blood flow distal to the stenosis compared to that in the absence of stenosis. It measures the ratio of the distal coronary pressure to aortic pressure under a hyperemic state. This index quantifies the severity of coronary artery stenosis by evaluating its functional impact on blood flow. Since the possibility of myocardial ischemia cannot be evaluated by the geometrical structure directly, FFR values give a more credible indication of the likelihood of ischemia resulted from the stenotic lesion. Generally, FFR is measured during a percutaneous intervention, which is an invasive procedure dealing with coronary artery disease^2,3^. FFR is an important clinical tool to guide intervention strategy when we manage intermediate stenosis. A 15-year follow-up study showed that the deferred percutaneous coronary intervention (PCI) after the safety evaluation by FFR for functionally nonsignificant stenosis demonstrated a lower rate (2.2%) of myocardial infarction than those who underwent revascularization (10%)^4^. However, such an invasive procedure poses certain risks to patients because of potential allergic reactions to vasodilators such as adenosine and possible complications during the prolonged procedure^5,6^. Thus, the risk of invasive procedures and both patients’ and physicians’ willingness to undergo such an examination would be very important concerns throughout the therapeutic courses.

Alternatively, FFR values can be derived without catheterization. Computational fluid dynamics (CFD) can simulate the pressure and velocity fields within coronary arteries non-invasively. The anatomical structures of the blood vessels can be reconstructed from coronary computed tomography angiography (CCTA)^7–12^ and coronary angiography (CAG)^3,8,13–16^ images. CCTA-derived FFR has been recognized as a helpful guide for appropriate use criteria for coronary revascularization by the American College of Cardiology^17^. To accurately simulate the flow field in vessels requires an adequate description of boundary conditions, which can be specified by calculating the subtended myocardium and applying allometric scaling laws to estimate the coronary blood flow or applying lumped parameters models to estimate microvascular resistance for the outlet conditions^11,12,18^. Some studies measured the pressure and flow and predicted the pulse pressure (systolic minus diastolic pressure) by fitting the two-element Windkessel model^19^. The time-varying pressure could help to simulate the deformability of the aorta and arterial walls^20,21^. However, measuring the pulsating aortic pressure requires inserting a pressure wire into the aorta. This defeats the purpose of deriving a noninvasive CCTA-based FFR instead of a direct FFR measurement. Consequently, most studies still assumed static blood pressure because of the inaccessibility to dynamic blood pressure. Previous works usually specified boundary conditions by utilizing the static blood pressure, estimating the blood flow rate according to the cardiovascular resistances^7,11^, or calculating the blood velocity using CAG frames^3,8,14,22,23^. The detailed setting of inlet and outlet boundary conditions is usually not explicitly described in the literature.

Besides FFR, the wall shear stress (WSS) induced by the coronary blood flow can also be a possible causative factor for high-risk plaque development^24,25^. Since high WSS facilitates the development of a greater plaque necrotic core and densifies the calcification progression^24^, the region of stenosis can deteriorate and lead to further atherosclerosis in the near future. Proximal WSS becomes an incremental prognostic index over FFR in predicting myocardium ischemia in recent years^26,27^.

Here, we aim to specify a CFD simulation protocol for CCTA-derived FFR with well-defined, patient-specific boundary conditions. Our method can take into account both the geometrical configuration of coronary arteries and the myocardium territory for coronary flow estimation. Our study did not simplify the inlet boundary condition as a constant inflow rate for every patient, nor did we need a dynamic computed tomography perfusion imaging that exposes patients to more radiation dose^28^. The outflow mass flow rate was estimated based on the myocardium territory of coronary arteries and Murray’s law to make the blood flow allocation more precise and raise the accuracy of diagnosis. This territory-based method of mass flow rate estimation can provide a patient-specific boundary condition estimation for the coronary artery simulation. With the boundary conditions derived from the territory-based method, FFR and WSS can be computed by the CFD simulation readily. The simulated FFR and WSS results can be used as indices for myocardium ischemia diagnosis and as guidance for treatment strategy.

## Methods

### Data description

Twenty patients with intermediate cardiovascular risk who underwent CCTA and subsequent CAG and FFR from a single institute were retrospectively collected in this study. Among them, a total of 25 diseased arteries with stenosis are included for analysis. The data characteristics per patient are summarized in Table 1. CCTA exams were performed on a 16-cm wide detector CT (Aquilion ONE; Toshiba Medical Systems Corporation) with standard coronary CT angiography protocol in our institute. In all patients, subsequent CAG was required clinically for further diagnosis or intervention and performed at our catheter lab (Allura Xper system; Philips Medical Systems). The FFR measurement was performed under the maximum achievable blood flow, which requires adenosine dosage to reach maximum hyperemia. The study is approved by Institutional Review Boards, and the TAIRB number is 201801039RINC.

**Table 1:** Clinical data and stenosis characteristics.

**Patients’ data** (*n*=20)	
Male, *n* (%)	12 (60%)
Age, years	$66.5 \pm 8.7$
Diabetes mellitus, *n* (%)	5 (25%)
Hypertension, *n* (%)	12 (60%)
Hyperlipidaemia, *n* (%)	12 (60%)
Smoking (current $+$ past), *n* (%)	5 (25%)
Patients with FFR $\le$ 0.80, *n* (%)	5 (25%)
**Vessel data** (*n*=25)	
**Stenosis characteristics and locations**	
Calcium score (mean $\pm$ SD)	343.6 $\pm$ 337.8
LAD, *n* (%)	16 (64%)
LCx, *n* (%)	5 (20%)
RCA, *n* (%)	4 (16%)
Vessels with FFR $\le$ 0.80, *n* (%)	5 (20%)

### Artery rendering

The ground truths of coronary artery centerlines and lumen used in this study were labeled by professional annotators with 7 and 2 years of experience in coronary artery processing, and the annotation was then modified and verified by cardiologists and radiologists in the core lab. The annotation was done on the zoomed cross-section of a coronary artery to ensure better fitness of the spline curve to the lumen. In case that blooming artifacts resulted from severely calcified plaque might hinder the discrimination of true lumen boundary^29^, coronary artery angiography was used as a reference to exam such regions with blooming artifacts.

The 3D images underwent the following preprocessing steps of coordinate rescaling, 3D Delaunay triangulation^30^, and meshing. The image coordinate must be scaled by the voxel dimensions along each axis to transform the CCTA coronary arteries from image coordinate to real anatomical coordinate (patient coordinate). The point cloud of lumen labels was reconstructed by 3D Delaunay triangulation^30^. The triangulated artery was then converted to mesh before being used in the CFD simulation. The grid has undergone the grid independence test to ensure the accuracy and consistency of the simulation.

Since the diameter of the coronary artery is in general about 1.5 to 5 mm, the element size was set to be about 0.05 mm. For the stenosis region and distal regions, the meshes were especially refined to capture detailed pressure variation. The average numbers of nodes and elements are about 5 million and 25 million in the mesh of a single artery. The maximum skewness cannot exceed 0.95 to make certain the mesh quality and simulation convergence. The meshing process costs about an hour for a single artery branch.

### CFD simulation

Additional to the artery rendering, the boundary condition calculation, fluid characteristic property setting, and the mesh generation process are all important to the FFR and WSS simulation.

#### Inlet boundary condition

The boundary conditions for each coronary artery were inlet pressure and outlet mass flow rate. The inlet pressure of the coronary arterial branch is very close to aortic pressure if there are no plaques at the proximal regions. Therefore, the inlet pressure for each arterial branch is represented by the average aortic pressure of each patient. The diastolic and systolic periods respectively account for two-thirds and one-third of a cardiac cycle. Consequently, the average aortic pressure is obtained by a weighted average of the diastolic and systolic pressure according to Eq. (1) as

1 $$\begin{aligned} ABP = \frac{1}{3}SBP + \frac{2}{3}DBP, \end{aligned}$$

where *ABP* denotes the average blood pressure (mmHg), *SBP* means systolic blood pressure (mmHg), and *DBP* means diastolic blood pressure (mmHg).

#### Outlet boundary condition

Next, the other boundary condition for the CFD simulation is the outlet mass flow rate. This study utilized the “Territory-based method” to estimate the outlet boundary conditions of coronary arteries. To evaluate the performance of the proposed territory-based method, the commonly used “Windkessel-model-based method” and the “Frame-count-based method” were applied to our dataset for comparison. For territory and Windkessel-based methods, the left ventricular myocardium territory can be a useful tool to calculate the total blood flow in the cardiac system and then to allocate blood flow into each coronary artery. According to the allometric scaling law^31–33^, the relationship between the mass of the left ventricular myocardium and the total required blood flow can be expressed as a power-law equation like

2 $$\begin{aligned} Q = a\, M_{myo}^b, \end{aligned}$$

where *a* denotes the characteristic constant of the cardiovascular system, *b* is the scaling exponent, $M_{myo}$ corresponds to the left ventricular mass (g), and *Q* is the volumetric flow rate in coronary arteries (ml/min). The values of coefficients $a=0.64$ (ml/min $\cdot$ g)^32^ and $b=0.75$^31,33^ were assumed in our simulation. The value of *a* was derived from the experiment and *b* was derived theoretically and validated by experimental observations. The mass flow rate is the multiplication of the volumetric flow rate and the blood density (1060 kg/${\mathrm m}^3$)^32^.

##### Territory-based method

**Figure 1:**
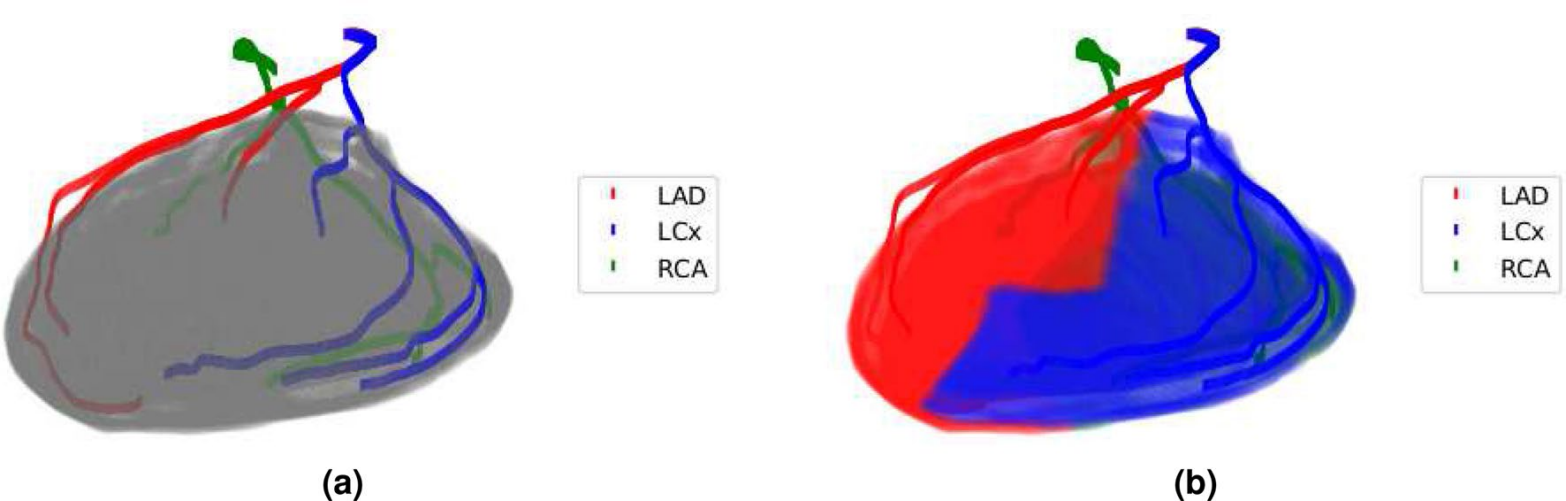
**(a)** LAD, LCx, RCA, and left ventricular myocardium are illustrated in red, blue, green, and gray, respectively. **(b)** The corresponding myocardium territory regions allocated to LAD, LCx, and RCA are painted in red, blue, green, as well.

The Territory-based method assigned each voxel of the myocardium to the nearest coronary artery after extracting the left ventricular myocardium from CCTA images. As shown in Fig. 1, there are three main coronary arteries, i.e., LAD, LCx, and RCA. Each of them governs part of the heart muscle. The concept of artery territory is similar to the drainage basin of a river. The catchment area belongs to a particular coronary branch. The larger a catchment area is, the more blood flow is needed to meet the nutritional and oxygen needs. Assuming oxygen is supplied by the nearest branch and each gram of myocardium has the same amount of nutrition needs, the territory segmentation can derive the allocation of blood flow in coronary arteries. By allotting the left ventricle myocardium voxels to LAD, LCx, and RCA, the amounts of myocardium voxels allocated to these arteries should be proportional to the blood flow rates^28^. Nevertheless, since we can only extract the main branches of coronary arteries from CCTA, small and trivial side branches invisible on CCTA images are neglected. To avoid the error caused by ignoring these trivial side branches, we applied Murray’s law to allocate the blood flow after bifurcation points^21,27,34^. This method is termed the Territory-based method because the estimation of the mass flow rate for the cardiovascular simulation is based on the myocardial territory that each coronary artery governs. Fractional flow reserve and wall shear stress can be derived from the simulation results and be used as indices for cardiac ischemia.

##### Windkessel-model-based method

To compare with the boundary conditions commonly used in other works, we also estimated the cardiovascular resistance and counted the CAG frames to calculate the outlet mass flow. First, the Windkessel model-based method analogized the pressure, flow rate, and resistance in the cardiovascular system to voltage, current, and resistance in an electric circuit^35^. The resistance and capacitance of each coronary artery branch depend on the location of plaques and the severity of stenosis. Since the patients are under rest state during the CCTA inspection, we refer to the parameters in Table 3 of Kim et al.’s work^21^ under rest state. These resistance values at rest state were further multiplied with the total coronary resistance index (TCRI) to derive the resistance values under hyperemic state^36^. Since the poor distensibility and inaccessibility of dynamic pressure information, the effects of capacitance are not considered here. The coronary arterial resistances (${R}_{a}$) of the main branch and side branches of LAD, LCx, and RCA are lumped in parallel. The blood flow in LAD, LCx, and RCA can be estimated to be inversely proportional to the overall resistance from the aorta to the distal end^21,37^. After rendering the artery from CCTA images, the mass flow rate derived from the allocation of Windkessel resistances was used as the outlet boundary condition to simulate the pressure field in coronary arteries.

##### Frame-count-based method

Another way to obtain the mass flow rate is to compute the flow velocity observed in CAG images by counting the number of cine frames. Thrombolysis in Myocardial Infarction (TIMI) frame count can be a useful tool for the calculation of velocity, and the subsequent mass flow rate^8,14,23^. The definition of frame count velocity is the time period during which the contrast agent passes from the beginning of the artery to the end divides the length of the artery in 3D CAG images. It is worth noting that since the coronary angiography machine shots 15 cine frames per second isochronously, the number of cine frames from the first to the last frames is directly proportional to the time for the blood flowing through the artery. However, the arteries visualized in a CAG image are the projection of a 3D artery structure onto a 2D plane, which cannot correctly reflect the actual arterial length and will underestimate the artery length at most projection angles. Because reconstructed CCTA images are 3D in nature, the length of an artery is not influenced by the projection angle. Therefore, CCTA images can also help to fix this problem, in addition to building the coronary vasculature. By registering corresponding feature points between CAG and CCTA images, the actual artery length can be computed more precisely. Finally, the mass flow rate was obtained by multiplying density, the frame count velocity, and the cross-sectional area. This is referred to as the Frame-count-based method hereafter.

#### Simulation details

The CFD simulation of coronary arteries was performed with ANSYS Fluent 2020 R1. The blood was assumed to be Newtonian fluid (viscosity $=$ 0.03 poise). The coronary arterial walls were assumed to be rigid in the simulation. As for the viscosity, although the hematocrit ratio can influence the viscosity of blood^38^, the variation range is small and the viscosity value could be assumed constant despite the pressure variation. The blood flow was set to be an incompressible fluid of density equal to 1060 kg/${ \mathrm m}^3$^39,40^. Usually, the simulation for a single artery branch could converge in about 500 iterations and the calculation time was 2 $\sim$ 3 h for a single branch.

#### FFR and WSS$_{\mathrm{prox}}$

After the computation, the pressure field and WSS on the arterial wall can be obtained. FFR is defined as the ratio of distal pressure ($$P_{d}$$) to aortic pressure ($$P_{a}$$), where $$P_{a}$$ is the pressure at the beginning of the artery, whose value is very close to the aortic pressure and $$P_{d}$$ is the pressure value at 5 mm succeeding a stenotic lesion. The definition of proximal WSS ($$\mathrm {{WSS}_{prox}}
$$) is the WSS value averaged over the circumference of the proximal third of the stenosis region^27^.

### Statistical analysis

Scatter plot and Bland-Altman plot will be used to examine the correlation and agreement between simulated FFR (FFR$_{s}$) and measured FFR (FFR$_m$) values. The continuous variables, such as the blood pressure or FFR values, are expressed as their mean values with a tolerance of the standard deviation (mean ± SD), hereafter. A per-vessel receiver operating characteristic (ROC) curve was applied to compare the performance of the above three methods. The curve was constructed by applying different discrimination thresholds to test the diagnostic ability of a binary classifier system. All the 25 blood vessels in this study are included. If the catheterized FFR value measured under maximum adenosine dosage is larger than the cutoff value of 0.80, patients may defer PCI treatment and resort to optimal medical treatment (OMT). The amount of adenosine usage may be different for each patient. The values of accuracy, sensitivity, and specificity are the classification results based on the threshold of 0.80. An FFR value under or equal to 0.80 is considered positive while an FFR value above 0.80 is considered negative.

## Results

### FFR simulation

After the preprocessing and CFD simulation, the pressure and velocity field distributions within each coronary artery were obtained. Subsequently, FFR values for different coronary arteries were calculated accordingly, and are denoted as ${\mathrm{FFR}}_{s}$. The values can be compared with the catheter measured FFR, which is denoted as ${\mathrm{FFR}}_{m}$. Subscripts *s* and *m* are added to FFR to represent the simulated and measured FFR values, respectively. The average proportions of LAD, LCx, and RCA myocardium territory in this study are 43.35%, 29.77%, and 26.87%, respectively.

**Figure 2:**
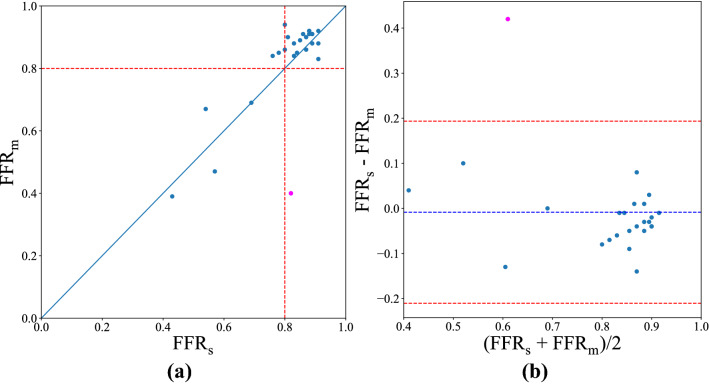
The measured FFR values (FFR$_{m}$) vs. simulated FFR values (FFR$_{s}$). **(a)** The scatter plot of simulated FFR and measured FFR. The red dashed line corresponds to the threshold of 0.8, which is the general threshold for severe stenosis and not severe stenosis. **(b)**The Bland–Altman plot. The red lines represent the 95% confidence interval (mean ± 1.96 SD) and the blue line means the average difference between simulated FFR and measured FFR.

The scatter plot shown in Fig. 2a displays the correlation and similarity between FFR$_{s}$ and FFR$_{m}$. The continuous variables are expressed as mean ± SD, hereafter. The FFR values derived from the Territory-based method and catheter measurements are 0.804±0.120 ($Q1 \sim Q3: 0.80 \sim 0.88$) and 0.812± 0.158 ($Q1 \sim Q3: 0.84\sim 0.91$) with the correlation coefficient equal to 0.758. Applying the FFR cutoff value of 0.8 to the Territory-based FFR$_s$ resulted in 4 true positives, 18 true negatives, 2 false positives, and 1 false negative out of 25 CCTA vessels with visible plaques. The overall accuracy, sensitivity, specificity, and precision are 88%, 80%, 90%, and 66.7%, respectively. The Bland-Altman plot in Fig. 2b examined the agreement between the simulated FFR and catheterized FFR values. No significant difference between FFR$_{s}$ and FFR$_{m}$ is found (p-value = 0.347). Furthermore, the discrepancy between measured and simulated FFRs is consistent with their averages, indicating the uniformity of variance. The baseline blood pressure without vasodilator at the guiding catheter tip of these 20 patients measured in this study was 93.65 ± 12.52 mmHg.

### Comparison of the stenosis significance assessment using different mass flow rate estimation methods

**Figure 3:**
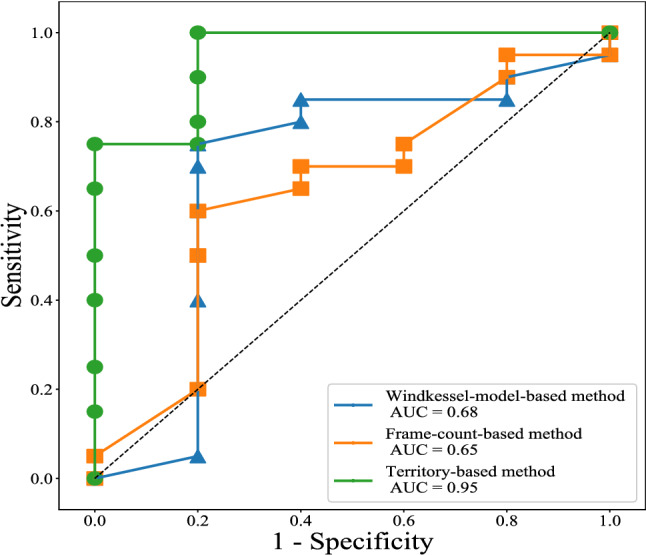
The receiver operating characteristic curve.

The Windkessel-model-based and Frame-count-based methods were applied to our dataset to estimate the outlet mass flow rates. The resulting FFR values were compared with those obtained by using the proposed Territory-based method. The accuracy, sensitivity, and specificity of classification results are shown in Table 2. The ROC curve in Fig. 3 compares the diagnosis ability of these three methods by the values of area under the curve (AUC). Territory-based method, Windkessl-model-based method, and Frame-count-based method have the values of AUC of 0.95, 0.68, and 0.65 respectively.

**Table 2:** Comparison of per vessel accuracy, sensitivity, and specificity at the threshold of 0.80. The asterisk denotes the best performance.

Methods	Accuracy	Sensitivity	Specificity
Territory-based	0.88*	0.90	0.80*
Windkessel-model-based	0.76	0.90	0.20
Frame-count-based	0.80	0.95*	0.20

### Wall shear stress

**Figure 4:**
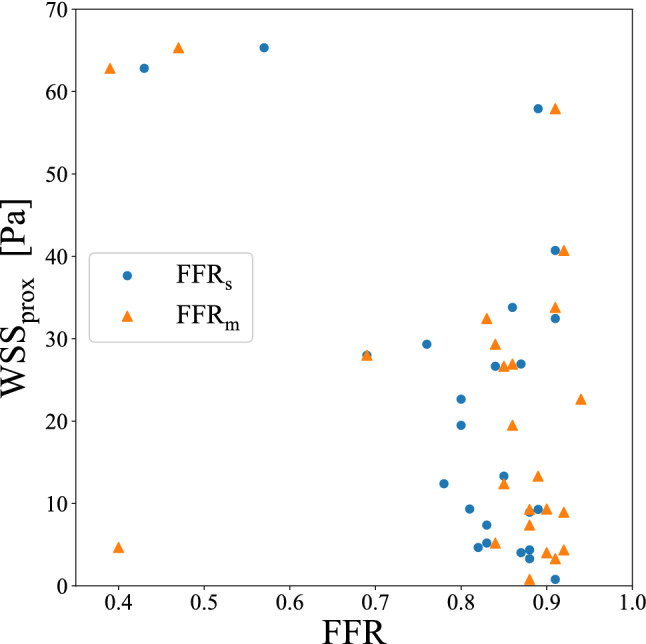
The relation between the proximal wall shear stress ($\mathrm {{WSS}_{prox}}$) vs. FFR.

**Figure 5:**
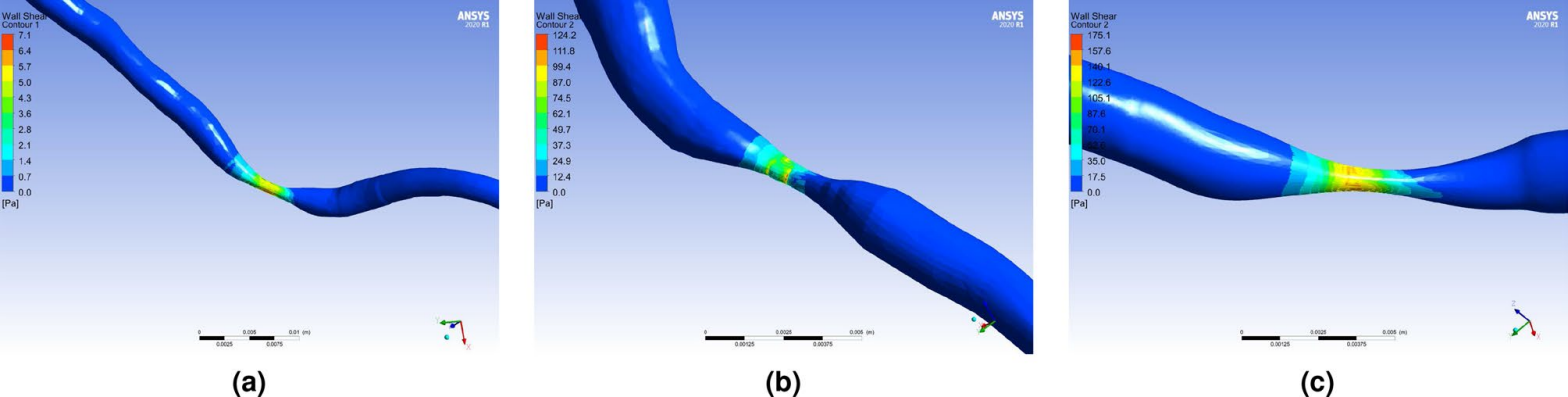
Color-coded wall shear stress for vessels with **(a)** high FFR and low $\mathrm {{WSS}_{prox}}$, **(b)** high FFR and high $\mathrm {{WSS}_{prox}}$, or **(c)** low FFR and high $\mathrm {{WSS}_{prox}}$.

Our research correlated the $\mathrm {{WSS}_{prox}}$ based on the CCTA image with both measured FFR$_m$ and simulated FFR$_s$. The results are shown in Fig. 4. The blue circles and orange triangles correspond to $\mathrm {{WSS}_{prox}}$ vs. FFR$_{s}$ and $\mathrm {{WSS}_{prox}}$ vs. FFR$_m$, respectively. The values of $\mathrm {{WSS}_{prox}}$ and FFR$_{s}$ show a better agreement since they are both derived from the CFD simulation. Severe plaques often lead to high $\mathrm {{WSS}_{prox}}$ and low FFR. On the other hand, non-severe stenosis often shows low $\mathrm {{WSS}_{prox}}$ and high FFR values. The orange triangles FFR$_{m}$ and blue circles FFR$_{s}$ are in general very consistent. An outlier is observed, which is further discussed in the Discussions section. Figure 5 shows some examples of color-coded wall shear stress distributions of stenotic regions. Subfigures (a) and (c) correspond to clinically mild and severe stenoses while subfigure (b) shows the stenosis that poses a higher myocardial risk.

## Discussions

### The effects of myocardium territory and mass flow rate on FFR simulation

The outlet boundary condition of mass flow rate plays an important role in the FFR simulation. The ratio of translesional pressure drop not only depends on the geometrical characteristics of the plaques but also other physiological factors. The same level of diameter or cross-sectional reduction could yield different FFR values in different coronary arteries. The phenomenon can be explained by the Windkessel model. If the resistances in the two vessels are the same, the vessel with more blood flow can yield a higher pressure drop, thereby a lower FFR value. Consequently, the mass flow rate in each vessel is a critical factor to be considered while interpreting the FFR simulation results. In view of the fact that the coronary mass flow rate is highly related to heart size and the myocardium territory proportions of the LAD, LCx, and RCA, it can vary significantly among individuals^41–43^. To better implement the CFD simulation, the mass flow rates for coronary arteries should be customized on a patient-specific basis. Hence, we assumed the blood flow needed for the oxygen and nutrient supply is proportional to the myocardium territory.

However, the underlying assumption of homogeneous oxygen and nutritional demands throughout the myocardium cannot always be met. In particular, for those with different pathological and physiological conditions, the oxygen consumption and coronary reactivity can distribute unevenly with a large variation throughout the myocardium^44–46^. Moreover, the flow in coronary arteries is also affected by vessel reactivity, thus extensive studies have been conducted on the analysis of relevant variables^32,47,48^. Of these variables, myocardial mass is definitely among the most important factors that affect the supply of blood flow^32^, and it is also among the variables which can be acquired on clinical coronary artery CT scans. Other factors are also very important, but it may be difficult to obtain reliable estimates from clinical CCTA scans. When the cardiovascular function degenerates, it is possible to redistribute blood flow from one region to another within tissue to accommodate any alterations in local metabolic needs. The coronary blood flow in the postischemic myocardium was reported to be unchanged even though the myocardial oxygen consumption was slightly higher than the control in a canine study^47^.

Schrauwen et al. reconstructed coronary bifurcations from CTA images and performed CFD simulation to obtain simulated FFR and WSS using different outlet boundary conditions^28^. Their work focused on the specification of the outflow ratios based on the diameters of daughter branches, volumes of the myocardium, or CTP-derived flow. Though the lumens of 10 patients were reconstructed, the simulated FFR values were never compared with actual measurements in their studies. On the other hand, our research applied patient-specific inlet and outlet boundary conditions that aim to yield FFR values close to CAG-measured FFR values. While only the simulated results for three main coronary arteries are shown here, our method can be readily extended to large branches. We followed Murray’s law to set the flow for side branches because they are usually invisible in CCTA images and it is impractical and inaccurate to allocate blood flow based on their myocardium perfusion. As described in the Patient selection of Schrauwen et al.’s work, only bifurcations with no other side branches visible on the CTA data were selected in their study.

The outlier, the magenta circle in Fig. 2a,b, has the smallest myocardium mass of 71.51 g among the 20 patients. Compared to the average myocardium mass of 114.31 ± 31.42 g, it is smaller than about 65% of the population. It was indicated that the exponent of 0.75 in the allometric scaling law only holds in the limit of organisms of infinite size^49^. Deviation from the value for small mammals was reported^49,50^. Therefore, the small myocardium can cause significant bias for the mass flow rate estimation. Applying the same scaling parameters to an underweight myocardium could lead to the underestimation of flow rate, thereby an exceptionally high FFR value. The outlier can also be attributed to an inhomogeneous distribution of blood flow throughout the myocardium. For patients with past myocardial ischemia or infarction history, the redistribution of blood flow may result in the deviation from the scaling law.

The Windkessel-model-based method did not take into account the physical and physiological differences among individuals. Our proposed Territory-based method, on the other hand, takes into consideration the heart size and coronary artery distribution in the myocardium; thus it results in a more accurate depiction of an individual’s blood flow. Besides, the Frame-count-based method relies on manual counting of the period of contrast that passes from the beginning to the end of the vessel in CAG images. Theoretically, this method should be the closest to reality, but the 2D projection angles and temporal resolution can influence the accuracy of length arterial calculation. For some projection views, some parts of the vessels are overlapping. So even if we registered the points on a 3D CCTA image, there can be bias for the vessel length calculation. Moreover, the CAG images were acquired at a rate of 15 fps. The poor temporal resolution also introduced errors in the estimation of flow rate. Apart from this, a few frames of a time delay could exist while employing human eyes to track the trajectory of the contrast agents.

LAD, LCx, and RCA supply different parts of myocardium territories; therefore, these three branches will be discussed separately. LAD usually governs the largest part of the myocardium and has the maximum mass flow rate. This can lead to a more serious consequence if myocardial infarction occurs. The proportion of LCx and RCA territories are not far off, so the mass flow rates are similar. The Windkessel-model-based method allocated the blood flow into LAD, LCx, and RCA based on the values of resistance. The lower the resistance is, the more the blood flow in the branch is. Different from the other two methods, the Frame-count-method estimated the blood flow velocity solely based on the transient changes in CAG images. It has lower LAD and LCx mass flow rates than the other two methods. This can attribute to errors caused by the miscalculation of the cine frame counts. Since the left coronary artery has more branches and bifurcation points, the movements of the contrast agent are difficult to track and foreshortening and vessel overlapping in CAG images also present challenges to identify the flow path than the RCA.

Furthermore, the allocation of myocardium territory helped the calculation of mass flow rate in each coronary artery for the simulation and indicated the risk of myocardial infarction for each region. When a coronary artery has a low FFR value, the territory of the target branch can be highlighted as the high ischemia risk zone. This may help cardiologists to make diagnostic decisions, especially about the clinical significance of distal vessel stenosis.

### Wall shear stress

The wall shear stress (WSS) induced by the coronary blood flow plays an important role in atherosclerosis. While FFR reflects the pressure variation perpendicular to the cross-sectional area of vessels, WSS represents the tangential force adjacent to lumen walls. Although the directions of WSS and pressure are different, these two factors are associated with each other. Since high WSS facilitates the development of a greater plaque necrotic core and densifies the calcification progression, proximal WSS becomes an incremental prognostic index in predicting myocardium ischemia in recent years^24,26,27^. Kumar et al.^27^ constructed Kaplan–Meier curves to compare vessel-related myocardial infarction rates between lesions with $\mathrm {{WSS}_{prox}}>4.71$ Pa and those with $\mathrm {{WSS}_{prox}}\le 4.71$ Pa over the course of three years. Lesions with a higher proximal wall shear stress were observed to show a significantly higher rate of myocardial infarction.

Except for the outlier, the $\mathrm {{WSS}_{prox}}$ values of the 5 clinically severe stenotic vessels (FFR $\le$ 0.8) in our study were significantly higher than 4.71 Pa. Even though the FFR values for the other 20 vessels were above the threshold value of 0.8, 16 of them have proximal wall stress greater than 4.71 Pa. This presents an increased risk for myocardial infarction. The region of stenosis can deteriorate and even lead to cardiac events in the future. Therefore, patients should seek medical treatments and continue to monitor the progression of stenosis.

### Performance comparison with previous simulation studies

The performance of stenosis severity estimation for our cases was improved by analyzing patient-specific blood flow allocation. Table 3 shows that our Territory-based method yields very high accuracy and sensitivity compared with other works. Previous CAG-based studies seem to yield simulated FFR values closer to catheterized FFR values^14,15,23^. This may attribute to the fact that FFR measurement procedures are performed during a CAG examination, thus patients’ physical and physiological conditions during CAG imaging and FFR catheterization are much closer than those during CCTA imaging. On the other hand, most CAG measurements were performed about 1-year after CCTA procedures, and patients’ physical conditions may have changed. Even though the CCTA-based FFR values showed slightly larger deviations from the catheterized ones, the primary objective of FFR assessment is to identify the functional significance of coronary stenosis. Consequently, the performance of classifying severe and not severe stenosis is of greater clinical significance.

**Table 3:** Performance comparison between the proposed Territory-based method and previous works.

Source	Image modality	No. of patients (vessels)	Accuracy	Sensitivity	Specificity	Difference (mean ± SD)
Territory-based method	CCTA	20 (25)	0.88	0.90	0.80	− 0.008 ± 0.103
Coenen et al. (2014)^11^	CCTA	106 (189)	0.75	0.88	0.65	− 0.04 ± 0.13
De Geer et al. (2015)^51^	CCTA	21 (23)	0.78	0.83	0.76	− 0.03 ± 0.15
Kurata et al. (2017)^52^	CCTA	21 (29)	0.93	1.00	0.87	−0.04 ± 0.08
Ko et al. (2017)^12^	CCTA	42 (78)	0.84	0.78	0.87	0.065 ± 0.137
Giannopoulos et al. (2018)^53^	CCTA	60 (73)	0.90	0.79	0.98	0.009 ± 0.108
Tu et al. (2014)^14^	CAG	68 (77)	0.88	0.78	0.93	0.00 ± 0.06
Tu et al. (2016)^15^	CAG	73 (84)	0.87	0.78	0.91	0.03 ± 0.068

The setting of boundary conditions in other works is usually not explicitly specified. Table 4 further compares the boundary condition settings of previous works listed in Table 3. The boundary conditions set in some CCTA-based simulations were the vascular resistance^11,51,52^, which corresponds to our Windkessel-model-based method. CAG-based simulations either assumed that the outlet flow is fully developed^14^ or derived coronary flow from frame counts, which fall into the same category as our Frame-count-based method^15^. However, some of the studies listed in Table 3 did not elaborate the setting of boundary conditions and cannot be compared with our method directly^12,53^.

**Table 4:** Comparison of boundary conditions.

Source	Boundary conditions
Territory-based method	Inlet: pressure; outlet: coronary flow (myocardium territory $+$ Murray’s law)
Coenen et al. (2014)^11^	Coronary flow (Murray’s law) $+$ vascular resistance
De Geer et al. (2015)^51^	Derived from lumped models of the heart and of the coronary circulation (not defined explicitly)
Kurata et al. (2017)^52^	Inlet: pressure; outlet: vascular resistance (not defined explicitly)
Ko et al. (2017)^12^	Derived from structural deformation of coronary lumen and aorta
Giannopoulos et al. (2018)^53^	Not defined explicitly
Tu et al. (2014)^14^	Inlet: pressure; outlet: fully developed outflow
Tu et al. (2016)^15^	Inlet: pressure; outlet: coronary flow (from CAG velocity)

It should be noted that the individual differences among patients can be significant, which can introduce considerable variations to flow rate and FFR estimation. To make the FFR simulation process simple while realistic, we focus on the hemodynamic impact of stenosis at a steady state while taking into account the variations in physical dimensions of each individual’s heart and the spatial distribution of artery vasculature. Other peripheral influences and the time-varying interactions caused by the left ventricle, aorta, capillaries, and veins will drastically increase computational complexity; therefore, these effects are beyond the scope of this study and are not considered in this work.

## Limitations

This study proposed the Territory-based method to assign the boundary condition of outlet flow rate on a patient-specific basis. It applied the allometric scaling law to estimate the coronary blood flow and hence the outlet boundary condition for each coronary artery. However, it may produce significant bias for patients with unusually small myocardium size and lead to inaccurate estimation of total coronary blood flow. As a result, the simulated FFR values will be overestimated.

Second, nitroglycerin (NTG) and adenosine are used for different examination needs clinically. FFR inspection uses adenosine to increase the diagnostic rate while CCTA uses NTG for examination safety and effectiveness^54–56^. NTG and adenosine take effects on different positions of the coronary artery. NTG is also known to affect the diameter of the perfused artery on CCTA^57^. There are some studies about the physiological effects of NTG and adenosine on coronary arteries. However, it seems that there is no head-to-head comparison between NTG and adenosine for FFR in the literature. Although there are studies on FFR with different doses of adenosine^58^, NTG is very rarely used in FFR inspection clinically, so there is no reliable or trusted conversion standard. Moreover, the CCTA image and FFR catheterization are performed at different times. The geometries of the coronary artery in this study are extracted from CCTA, but the simulation results were evaluated by catheterized FFR. If patients’ physical conditions have experienced notable changes during this time period, the simulated FFR value will deviate from measured values.

Third, the estimation of total blood flow is based on the assumption of homogeneous oxygen and nutritional demands throughout the myocardium. Nevertheless, the tissues with different pathological and physiological conditions might have heterogeneous oxygen and nutritional consumption. The assumption may slightly overestimate the total blood flow. Furthermore, there are neither precise mass flow rate measurement data nor information of myocardial ischemia to prove our estimation. We can only use the catheterized FFR to assess the severity of the cardiovascular disease.

Last, the simulation results may be biased by the assumption of Newtonian fluid and rigid arterial walls. The Newtonian and non-Newtonian blood models did not show a great difference in flow patterns, but the non-Newtonian model can cause a significant increase in wall shear stress distributions^27^. This may influence the accuracy of wall shear stress simulation results. Moreover, the assumption of rigid arterial walls does not account for great curvature change or the motion of coronary arteries during the cardiac cycle^39^. The impacts of heart motion and plaque calcification were also ignored in this study.

## Conclusion

We proposed the Territory-based method that utilized myocardium territory allocation and Murray’s law to estimate the coronary blood flow rate to accurately simulate the pressure field within coronary arteries with adequate boundary conditions. In addition to accounting for the dimension variation among different individual’s myocardium, our method also considered spatial distribution of coronary vasculature and designated blood flow accordingly. Arteries with larger diameters usually have lower arterial resistance and more blood flow. This is consistent with the blood flow allocated by the Territory-based method. Plaques and stenosis blocking blood flow can cause a large pressure drop and a low FFR value. The quantitative, patient-specific estimation of flow rate yielded the best performance of separating severe and non-severe coronary stenosis, compared to conventional Windkessel-based and Frame-count-based methods. Additional to a low FFR, a high wall shear stress has been linked to enhanced plaque vulnerability. It was shown that high proximal wall shear stress is associated with an increased risk of ischemia. Therefore, attention must be paid to lesions with high proximal wall shear stress even if their FFR values are high.

## Data Availability

This study was approved by the Institutional Review Board (IRB) of National Taiwan University Hospital under TAIRB No. 201801039RINC. The clinical data are fully anonymized and the experiments were performed by a qualified cardiologist with good clinical laboratory practice in accordance with the relevant guidelines and regulations. The IRB granted a waiver of informed consent for the retrospective study of participant’s imaging data between January 2001 and June 2017 (2 out of 20 patients). Informed consent for all human subjects who participated after July 2017 (18 out of 20 patients) was obtained.
